# Percutaneous pulmonary valve implantation in a patient with congenitally corrected transposition of the great arteries: a case report

**DOI:** 10.1186/s13256-024-04383-9

**Published:** 2024-02-21

**Authors:** Michal Kapalka, Michal Galeczka, Michal Krawiec, Roland Fiszer

**Affiliations:** 1 Student Scientific Association at Department of Paediatric Cardiology and Congenital Heart Defects, Faculty of Medical Sciences in Zabrze, Medical University of Silesia in Katowice, Zabrze, Poland; 2 Department of Paediatric Cardiology and Congenital Heart Defects, Faculty of Medical Sciences in Zabrze, Silesian Center for Heart Diseases, Medical University of Silesia in Katowice, Curie-Sklodowskiej 9 Street, 41-800 Zabrze, Poland

**Keywords:** Congenitally corrected transposition of great arteries, Percutaneous pulmonary valve implantation, Melody valve, Coronary artery

## Abstract

**Background:**

Percutaneous pulmonary valve implantation has become an attractive method of dysfunctional right ventricle outflow tract treatment.

**Case presentation:**

We describe a unique case of a 20-year-old Caucasian male patient with a complex cyanotic heart defect, namely pulmonary atresia, with congenitally corrected transposition of the great arteries and ventricular septal defect after Rastelli-like surgery at the age of 5 years with homograft use. At the age of 20 years, the patient needed percutaneous pulmonary valve implantation owing to homograft dysfunction. Despite unusual course of the coronary arteries, balloon testing in the landing zone of the right ventricle outflow tract excluded potential coronary artery compression. Then, after presentation, a Melody valve was implanted successfully in the pulmonary valve position. The 8-year follow-up was uneventful.

**Conclusion:**

This is likely the first description of a percutaneous pulmonary valve implantation in such anatomy. Such a procedure is feasible; however, it requires exceptional caution owing to the anomalous coronary arteries course, which can be the reason for their compression.

**Supplementary Information:**

The online version contains supplementary material available at 10.1186/s13256-024-04383-9.

## Background

Percutaneous pulmonary valve implantation (PPVI) has become an attractive method of dysfunctional right ventricle outflow tract (RVOT) treatment [1]. We describe a unique case of a patient with a complex cyanotic heart defect, namely pulmonary atresia (PA), with congenitally corrected transposition of the great arteries (ccTGA) and ventricular septal defect (VSD) in whom PPVI was performed. Such a case has not been reported thus far.

## Case presentation

A 20-year-old Caucasian male patient with complex cyanotic heart defects, namely, ccTGA, PA, and VSD, underwent left-sided Blalock–Taussig–Thomas shunt palliation at the second day of life, followed by a correction at the age of 5 years. The surgery included ventricular septal defect (VSD) patch closure and subpulmonary outflow tract reconstruction with a 17-mm homograft (Rastelli-type procedure). He was readmitted at 20 years old, with no symptoms in anamnesis and with good exercise tolerance. A loud systolic murmur with a thrill and soft diastolic murmur were found upon physical examination. Echocardiography revealed good function of the systemic ventricle—morphologic right ventricle (mRV) and homograft obstruction with a maximum and mean gradient of 78 mmHg and 45 mmHg, respectively, as well as its' moderate regurgitation. The peak-to-peak gradient of 82 mmHg through the narrowed-to-10-mm homograft was measured in the diagnostic catheterization and the patient qualified for PPVI.

**Fig. 1:**
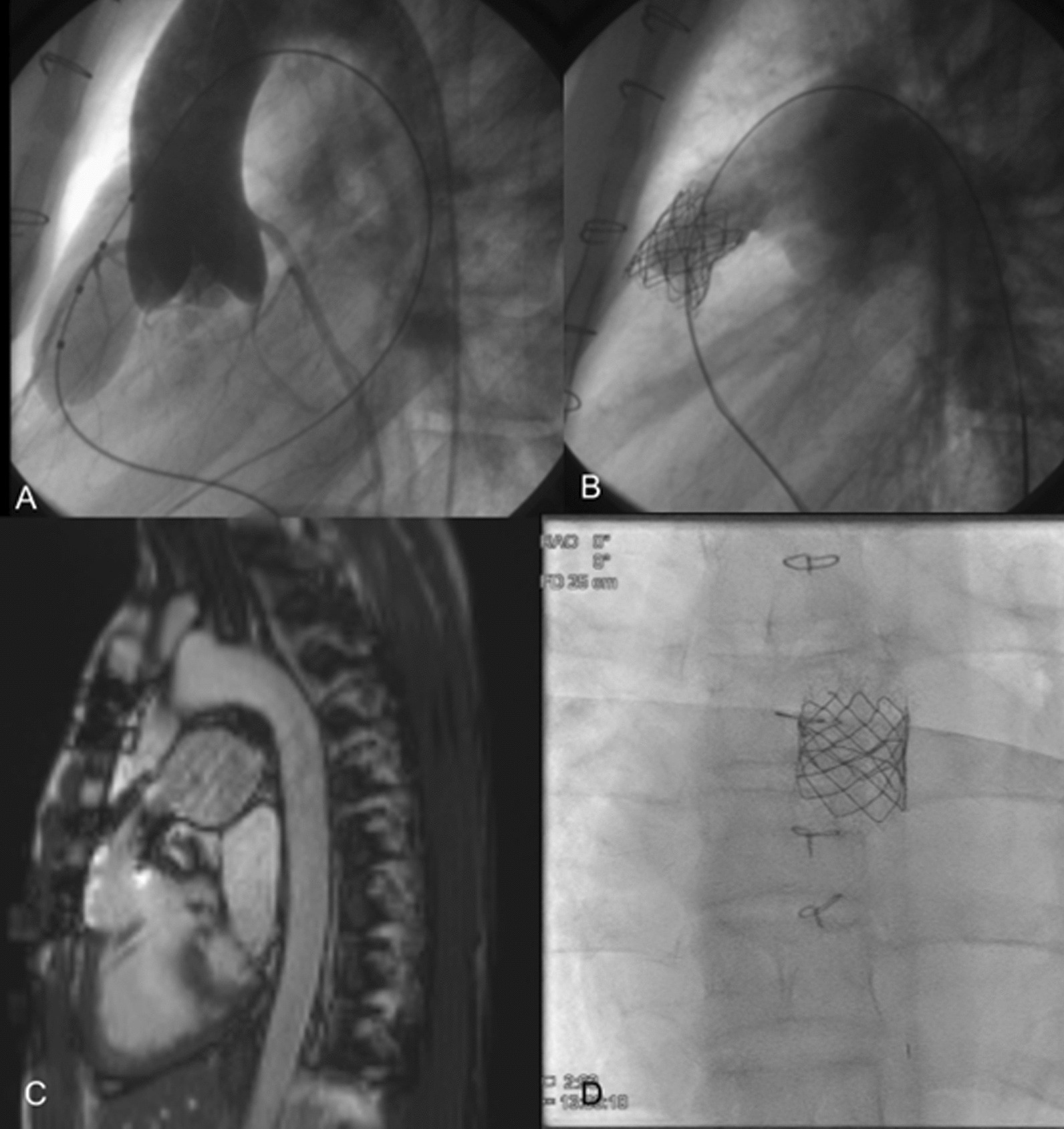
**A** Aortography performed during 20-mm Mullins-X balloon inflation in the potential valve landing zone in lateral projection shows no coronary artery compression. **B** Angiography performed after successful implantation of AndraStent and 18-mm Melody valve shows good effect of the procedure with trace of a regurgitation. **C** Magnetic resonance imaging performed 8 years after the procedure shows good performance of the conduit with no regurgitation, no subpulmonary valve enlargement, and preserved subpulmonary ventricle function (EF of 48%). **D** Fluoroscopy 7 years after implantation of the Melody valve shows small, nonsignificant stent fractures with preserved stent and valve structure

Nonselective coronarography was performed to exclude the possibility of coronary artery (CA) compression assessed by aortography with a 20-mm Mullins-X balloon placed in the potential valve landing zone (Fig. 1A). The test revealed that the right coronary artery (RCA) originated from posterolateral sinus and the left anterior descending artery (LAD), with the circumflex artery (Cx), originated from medial sinus separately. Despite the short distance between the Cx origin and the homograft valve annulus, coronary artery compression was not an issue.

The 35-mm AndraStent XXL (AndraMed GmbH, Reutlingen, Germany) was implanted into the homograft on a 20-mm balloon-in-balloon (BIB). Then, an 18-mm Melody valve (Medtronic Inc., MN) on an 20-mm BIB was expanded (Fig. 1B). Because of the residual peak-to-peak gradient of 30 mmHg, the 22 mm Mullins-X balloon was used for postdilatation, and the final gradient dropped to 15 mmHg (see Additional file 1: Video S1, S2; Additional file 2: Video S3, S4; Additional file 3: Video S5). In the 8-year follow-up, the patient remained asymptomatic with a mean residual gradient through the Melody valve of 22 mmHg on checkup echocardiography. Ejection fraction of the systemic ventricle was assessed to be 39% by magnetic resonance imaging (Fig. 1C). Single and insignificant fractures of the Melody valve stent (Cheatham-Platinum stent) are observed on fluoroscopy (Fig. 1D). No late complications, such as infective endocarditis, have been observed.

## Discussion and conclusion

Congenitally corrected TGA with VSD and PA can be surgically treated by closing the VSD and placing mLV-to-pulmonary artery conduit in some cases [2]. Every implanted homograft carries the risk of future dysfunction, mostly due to its calcification and stenosis. After the Rastelli procedure, 51% of patients had reoperations owing to stenosis and insufficiency of the extracardiac conduit [3]. In patients with RV to pulmonary artery conduit stenosis, transcatheter valve implantation in the pulmonary position can be performed effectively [4]. A case series of a PPVI in two patients with ccTGA after Senning surgery was reported [5].

During PPVI, special attention should be paid to the risk of compression of the coronary arteries (CA). Morray *et al*. reported coronary artery compression in 4.7% of patients observed on preimplant testing [6]. Examined patients had various heart defects, and the majority (71%) of coronary artery compressions were noted in patients with an abnormal course [6]. Haas *et al*. reported abnormal coronary arteries in 32.8% of patients with dysfunctional right ventricle outflow tract [7].

Exceptional attention should be paid in cases of PPVI in patients with ccTGA, as CA origins are malpositioned, which is directly associated with aortopulmonary rotation [8]. PV is often localized between the ascending aorta and right atrium; therefore, CA may course closely to the potential valve implantation landing zone.

Stent fractures are a common adverse effect after Melody PPVI and occur in 25% of patients [9]. Stent fractures that do not affect its structure are not significant complications; however, the unusual position of the RVOT in patients with ccTGA might result in more frequent occurrence of stent fractures. Despite those precautions, given the correct anatomical foundation, PPVI in patients with ccTGA can be safely performed.

PPVI can be safely performed in patients with ccTGA after Rastelli-like repair. However, it requires exceptional caution due to the anomalous CA course, which can be reason for CA compression.

### Supplementary Information

**Additional file 1: Video** **S1**, **S2.** Ventriculography at the level of homograft.

**Additional file 2: Video S3**, **S4.** Coronary artery testing.

**Additional file 3: Video S5.** Final arteriography after Melody valve implantation.

## Data Availability

Available on demand.

## Abbreviations

PPVI: Percutaneous pulmonary valve implantation
RVOT: Right ventricle outflow tract
PA: Pulmonary artery
ccTGA: Congenitally corrected transposition of great arteries
VSD: Ventricular septal defect
CA: Coronary artery
RCA: Right coronary artery
LAD: Left anterior descending artery
Cx: Circumflex artery
